# ZnO Luminescence and scintillation studied via photoexcitation, X-ray excitation, and gamma-induced positron spectroscopy

**DOI:** 10.1038/srep31238

**Published:** 2016-08-23

**Authors:** J. Ji, A. M. Colosimo, W. Anwand, L. A. Boatner, A. Wagner, P. S. Stepanov, T. T. Trinh, M. O. Liedke, R. Krause-Rehberg, T. E. Cowan, F. A. Selim

**Affiliations:** 1 Department of Physics and Astronomy, Bowling Green State University, Bowling Green, Ohio 43403, USA; 2 Institute of Radiation Physics, Helmholtz-Zentrum Dresden-Rossendorf, Bautzner Landstr. 400, 01328 Dresden, Germany; 3 Materials Science and Technology Division, Oak Ridge National Laboratory, Oak Ridge, Tennessee 37831, USA; 4 Center for Photochemical Sciences, Bowling Green State University, Bowling Green, Ohio 43403, USA; 5 Technische Universität Dresden, 01062 Dresden, Germany; 6 Martin-Luther University, Department of Physics, 06099 Halle, Germany

## Abstract

The luminescence and scintillation properties of ZnO single crystals were studied by photoluminescence and X-ray-induced luminescence (XRIL) techniques. XRIL allowed a direct comparison to be made between the near-band emission (NBE) and trap emissions providing insight into the carrier recombination efficiency in the ZnO crystals. It also provided bulk luminescence measurements that were not affected by surface states. The origin of a green emission, the dominant trap emission in ZnO, was then investigated by gamma-induced positron spectroscopy (GIPS) - a unique defect spectroscopy method that enables positron lifetime measurements to be made for a sample without contributions from positron annihilation in the source materials. The measurements showed a single positron decay curve with a 175 ps lifetime component that was attributed to Zn vacancies passivated by hydrogen. Both oxygen vacancies and hydrogen-decorated Zn vacancies were suggested to contribute to the green emission. By combining scintillation measurements with XRIL, the fast scintillation in ZnO crystals was found to be strongly correlated with the ratio between the defect luminescence and NBE. This study reports the first application of GIPS to semiconductors, and it reveals the great benefits of the XRIL technique for the study of emission and scintillation properties of materials.

ZnO, a wide-gap semiconductor with an exciton binding energy of 60 meV at room temperature, is an attractive material for many existing and future applications such as gas sensors, varistors, light-emitting diodes (LEDs), transparent electrodes, and scintillation detectors [see e.g.]^1^,^2^,^3^,^4^,^5^. The recent advancement in ZnO nanostructures has also extended its applications to new fields^6^,^7^,^8^,^9^. Despite decades of research, the control and detailed understanding of intrinsic defects in ZnO and their related emissions, which are crucial for many future applications, are still unresolved issues^10^,^11^,^12^,^13^. The continued development of ZnO-based optoelectronics and scintillation devices – in particular-requires an in-depth understanding of the microscopic origin of all the luminescence features in ZnO. The assignment of visible luminescence in ZnO, in particular, is still a significant controversial issue. Zinc vacancies, oxygen vacancies, copper impurities, zinc interstitials and anti-site defects have all been suggested as possible origins of the green luminescence in ZnO single crystals - with a number of publications supporting each possibility^11^,^12^,^13^,^14^,^15^. It is also important to acknowledge that the green luminescence is very broad which suggests that there are contributions from different origins. In this work, we apply X-ray-induced luminescence (XRIL) and photoluminescence (PL) spectroscopies to obtain new insight into the luminescence from high-quality ZnO single crystals. Our newly developed XRIL spectrometer^16^,^17^ provides a useful tool to simultaneously monitor most ZnO emissions from 200 to 800 nm and to acquire information about the relative intensities of luminescence peaks and their related origins - information that is not available from traditional PL measurements like those shown in Fig. 1 and discussed below. The principle of XRIL (Fig. 2) is based on the following: The absorption of X-rays in semiconductors leads to the generation of a number of free and bound electrons and holes, which may recombine to give near-band emission or transfer their energy to luminescence centers thereby inducing defect luminescence. Thus, by using X-rays for excitation and recording the emission from the crystal as a function of wavelength, we can collect the NBE and defect luminescence simultaneously and measure the ratio between their intensities. X-rays also penetrate through the entire sample thickness, and thus, the measurements are not affected by the surface states, which is often the case in PL or cathode-luminescence (CL) measurements. This makes XRIL a unique and superior technique for luminescence studies in bulk materials.

Defects in ZnO single crystals were populated by relevant thermal treatments, and their emissions were recorded. XRIL spectra are dominated by the 520 nm green emission, and the results showed that the relative emission between the NBE and defect luminescence strongly depends on the annealing atmosphere. To investigate the presence and nature of Zn vacancies, positron annihilation lifetime spectroscopy (PALS) can be performed, since it represents a sensitive method for measuring cation vacancies in semiconductors^18^. However, PALS measurements in ZnO have been a subject of discrepancies and associated intense debate^19^,^20^. Because of these factors, in his work we have performed PALS using gamma-induced positron spectroscopy (GIPS)^21^,^22^, a unique method that provides a positron decay curve that is free from background or source contributions^23^. Accordingly, this aproach should provide accurate measurements of positron lifetimes in ZnO. Standard PALS measurements in bulk materials are often performed using a Na-22 positron source (e.g., NaCl deposited on a thin foil of Kapton, Ni or Al), which induces about a 10% contribution to the measured spectrum from positron annihilation in the foil, NaCl - and sometimes at the interface between the sample and source. In GIPS, high-energy γ-rays directly produce positrons inside the sample^24^ - completely eliminating unwanted contributions from positron annihilation in either the source or cladding materials and, thereby, leading to more accurate measurements of positron lifetimes in general. This method also probes the entire volume of the crystal. Standard PAS techniques have been extensively used to study defects in metals and semiconductors, however GIPS facilities have been recently developed but were only used previously for the investigation of liquids and structural engineering materials^22^,^25^,^26^,^27^,^28^. A precise pulse structure of the bremsstrahlung radiation is needed that has been only realized at the ELBE (Electron Linac with high Brilliance and low Emittance) facility, at the Helmholtz-Zentrum Dresden-Rossendorf (HZDR). The present work represents the first application of GIPS to semiconductors and oxides. GIPS measurements on the ZnO crystals under investigation in this work show a single positron decay curve and yield the same lifetime value of 175 ps for all of the samples. This can be attributed to Zn vacancies that are passivated by hydrogen. Early measurements have reported bulk positron lifetimes of 170 ps in ZnO^19^. However, several subsequent measurements and calculations [see e.g.]^20^ have challenged this claim as discussed below and have shown that the 175 ps is, in fact, associated with hydrogen in Zn vacancies and that the bulk lifetime value is about 154 ps.

Interest in ZnO as a scintillator is stimulated by the very fast sub-nanosecond decay times of the free and bound excitons^29^. Ga-doped ZnO - in particular - has been investigated as a potential scintillation detector^30^,^31^,^32^,^33^,^34^, with most of the studies being carried out on thin films, powders, and ceramics, and only a few^35^,^36^,^37^ investigations have considered Ga-doped ZnO single crystals. To gain more understanding about the scintillation mechanism of ZnO, we have examined the scintillation signal from un-doped ZnO single crystals and have correlated this signal with the luminescence properties. By combining XRIL with scintillation measurements on ZnO crystals, we have demonstrated a strong dependence of the fast scintillation signal on the ratio between NBE and the defect luminescence.

## Results and Discussion

**Figure 1:**
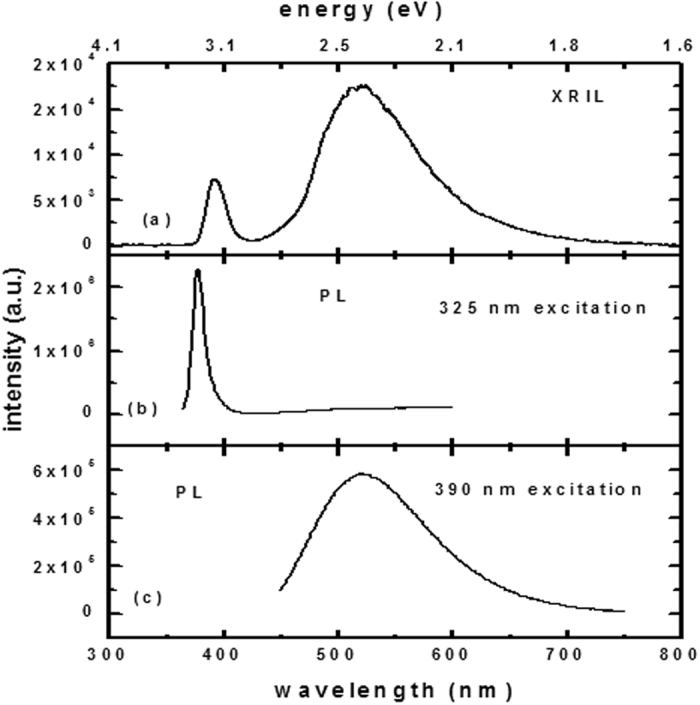
Luminescence spectra of ZnO single crystals grown by CVD and measured using: (**a**) X-ray excitation, (**b**) and (**c**) photo-excitation.

XRIL and PL spectra of an as-grown ZnO single crystal measured at room temperature are shown in Fig. 1(a–c). By using X-rays as the excitation source (Fig. 1(a)), two emission peaks are observed at 390 nm (3.17 eV) and 520 nm (2.44 eV). The first peak is observed at a lower energy than the near-band emission (NBE), which corresponds to band-to-band transitions and involves free excitons, donor acceptor pairs, and excitons bound to acceptors and donors and their electron satellites^29^. The peak value should be around 3.33 and 3.38 eV. The lower value observed here in the XRIL spectra is consistent with the NBE observed in room-temperature PL measurements that are commonly dominated by the longitudinal-optical phonon (LO-phonon) emission arising from the annihilation of free excitons. The second peak at 2.44 eV is attributed to defect luminescence in ZnO and will be discussed below. Photo-excitation with 325 nm light at room temperature (Fig. 1(b)) only reveals the NBE as shifted to 378 nm (3.28 eV). This peak is in agreement with most of the reported PL measurements at room temperature; it is attributed to annihilation of the LO-phonon-assisted exciton^30^ as explained above. It should be noted that the NBE peak position in XRIL is 0.11 eV lower than the NBE peak in PL, which may be due to the emission from higher order LO-phonon-assisted excitons. In the PL measurements, the defect luminescence peak at 2.44 eV cannot be seen with 325 nm excitation (Fig. 1b), however it is detected with the 390 nm excitation wavelength (1c). This definitely shows that XRIL has some advantages over PL because it allows a direct comparison to be made between the intensity of NBE and the defect luminescence in the crystals. Cathodo-luminescence (CL) can also provide a comparison between different luminescence centers, however XRIL is more advantageous for bulk studies because the thickness that CL can probe is limited by the range of electrons in the materials - while XRIL can probe the entire thickness of the sample. Moreover, the high-energy electrons in CL required for bulk measurements may induce structural defects in the sample, which is not the case with XRIL. An important advantage of XRIL over PL and CL is that the measurements are not affected by the surface layers because X-rays penetrate through the entire the thickness of the crystal. In PL, the 325 nm excitation light is absorbed in the first layers of ZnO because of its high absorbance in the UV region - making the PL signal limited to the surface and subsurface layers – i.e., strongly influenced by the surface conditions. As mentioned above, the surface layers may contribute in the CL measurements because of the limited range of electrons. However, the CL technique is especially useful for surface and depth-resolved measurements.

**Figure 3:**
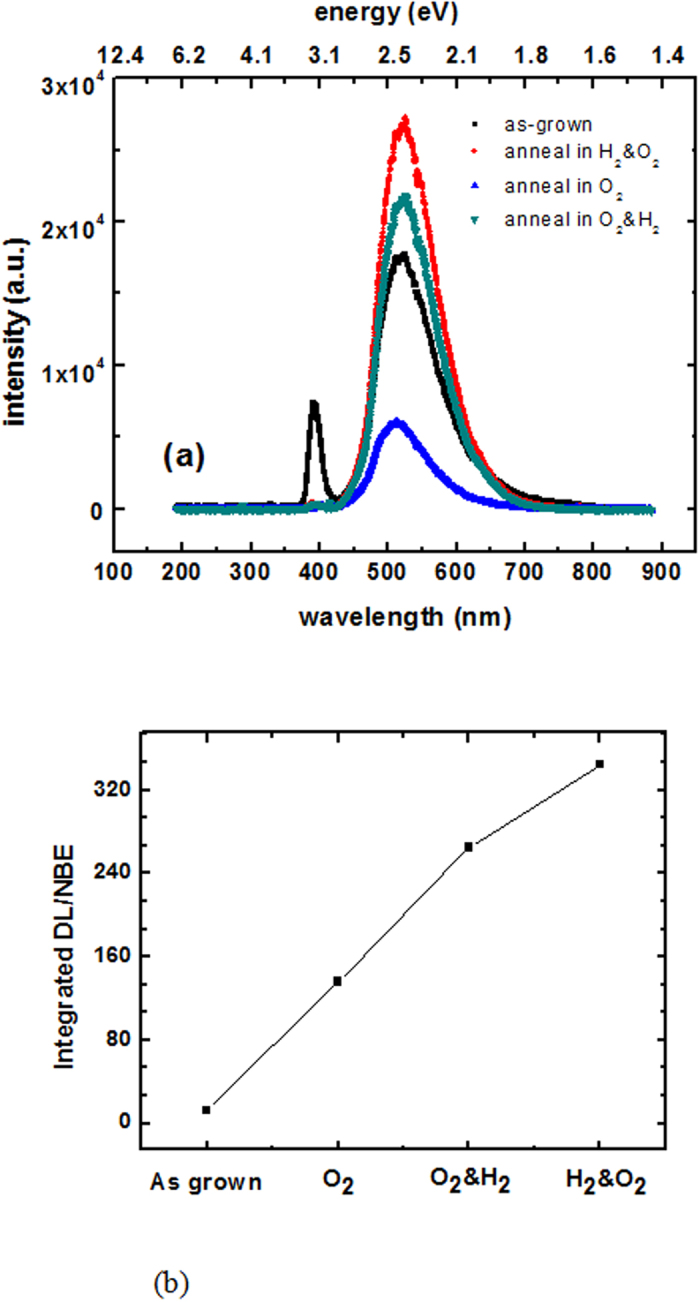
(**a**) XRIL spectra of as-grown and annealed ZnO single crystals. (**b**) Ratio of the defect luminescence to the NBE intensity for as-grown and annealed single crystals.

**Figure 4:**
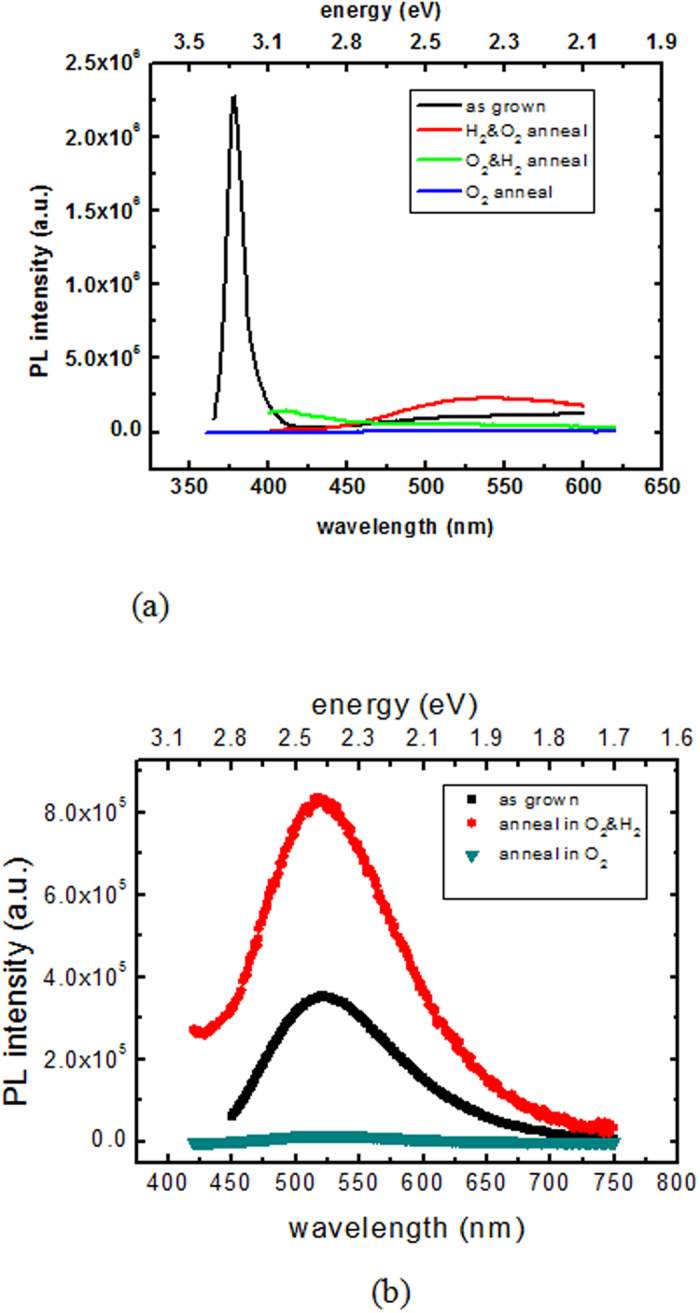
PL spectra of as-grown and annealed ZnO single crystals: (**a**) 325 nm excitation, (**b**) 390 nm excitation.

The deep-level emission at 520 nm (2.44 eV) in the XRIL and PL spectra is associated with native defects in ZnO. To study the origin of this emission at 2.44 eV, a number of ZnO single crystals were annealed in various atmospheres to populate or modify different types of defects. Fig. 3(a) shows the XRIL spectra for ZnO samples after each thermal treatment. The NBE emission was suppressed for all of the samples after annealing. With respect to the 520 nm (2.44 eV) emission, its intensity significantly decreased after the O_2_ anneal and increased after annealing in O_2_/H_2_ or H_2_/O_2_ atmospheres to a value that was even higher than that of the as-grown sample. This suggests that oxygen vacancies contribute to the 2.44 eV emission, since an O_2_ anneal would reduce the number of oxygen vacancies^15^. The ratio between the defect emission and NBE intensities was calculated for each sample and is displayed in Fig. 3(b). Its value for the as-grown sample is 12.7 and this value substantially increases after annealing due to the increase in the defect luminescence and/or the suppression of NBE. Although an O_2_ anneal fills the oxygen vacancies and decreases the green luminescence, it also dramatically suppresses the NBE - probably due to the decrease in donor concentrations as a consequence of the decrease of hydrogen impurities. Figure 4 (a,b) compare the room-temperature PL spectra for the as-grown and annealed samples using 325 and 390 nm excitation respectively. It can be seen that the NBE in the PL spectra was suppressed in all of the annealed samples, which is in agreement with the XRIL measurements. The change in the PL emission intensity after each anneal is also consistent with the XRIL spectra. The major advantage of XRIL over PL is that it allows one to measure the relative emission between NBE and the defect luminescence and thereby acquire information on the carrier recombination efficiency and trap density in the crystal. These measurements indicate the possibility of tuning the luminescence by appropriate thermal treatments. The ability to control defects and the ratio between NBE and trap emissions is key to optimizing the properties and performance of ZnO in various technological applications. In fact, ZnO is expected to be the most efficient light-emitting diode (LED) because of its 60 meV exciton binding emission. However its application to LEDs has been hindered by the failure to produce p-type material due to the lack of control of native defects and the absence of an understanding of the origin of the defect luminescence. Thus, the ability to monitor and control defects and luminescence is central to the successful development of ZnO light-emitting devices.

**Figure 5:**
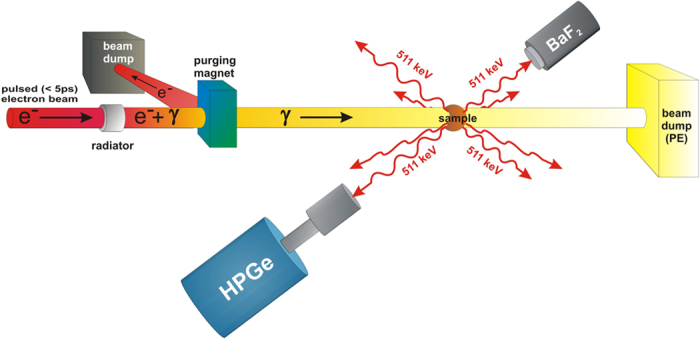
A schematic diagram of the GIPS set-up.

**Figure 6:**
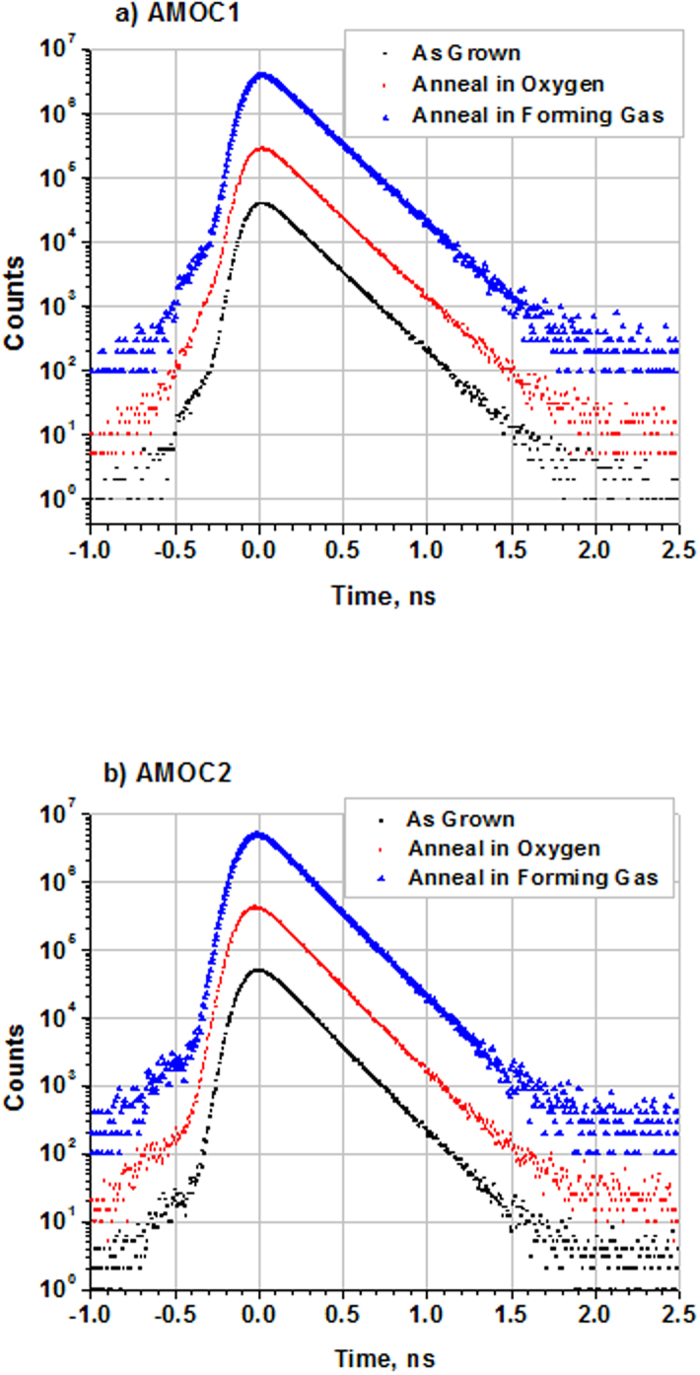
PALS spectra of ZnO single crystals measured by GIPS: (**a**) AMOC1, (**b**) AMOC2.

The deep-level emission peak is somehow much narrower than the deep emission peak commonly observed for ZnO - reflecting a decreased contribution from the red emission at 580 nm^15^. To investigate defects in these samples, PALS measurements using the GIPS apparatus (Fig. 5) were carried out for both the as-grown and annealed crystals. Figure 6 displays the lifetime spectra for as-grown, O_2_-, and H_2_/O_2_-anneal samples - as obtained using the AMOC1 and AMOC2 detection systems. The two AMOC data sets show similar positron decay curves for the three samples. Each lifetime spectrum was analyzed as the sum of exponential decay components convoluted with the Gaussian resolution function of the spectrometer. As is obvious from Fig. 6, all of the spectra show a curve with a single lifetime component. Table 1 displays the resolution function, positron lifetime value, and its standard deviations for each sample from the AMOC1 and AMOC2 systems. The average positron lifetime value from AMOC1 and AMOC2 is 175 ps, a value that is much higher than the 154 ps positron lifetime value obtained from annihilation in bulk ZnO as predicted by theory^20^. However, it is in agreement with the lifetime value for Zn vacancies passivated by number of hydrogen ions^20^,^38^,^39^,^40^,^41^. The CVT growth may lead to a high concentration of hydrogen and the resulting complete passivation of Zn vacancies by hydrogen. The presence of a single lifetime component indicates a saturation of positron trapping at the hydrogen-decorated Zn vacancies. Annealing in O_2_ or H_2_/O_2_ did not affect the positron lifetime spectrum - confirming the saturation of positron trapping. Oxygen vacancies cannot be seen by positrons because of their positive charge state; therefore, the presence of oxygen vacancies in the samples cannot be excluded based on our positron lifetime measurements. Annealing probably modified the amount of oxygen vacancies and the charge-carrier concentrations associated with hydrogen impurities, which in turn, affects both the NBE and DL results. Annealing may also change the amount of Zn vacancies passivated by hydrogen or the number of hydrogen ions that fill the Zn vacancy. However this effect could not be seen by PALS. The obvious conclusion from the PALS data is that Zn vacancies passivated by hydrogen (and not isolated Zn vacancies) are the main contributors to the 2.44 eV broad emission in ZnO.

**Figure 7:**
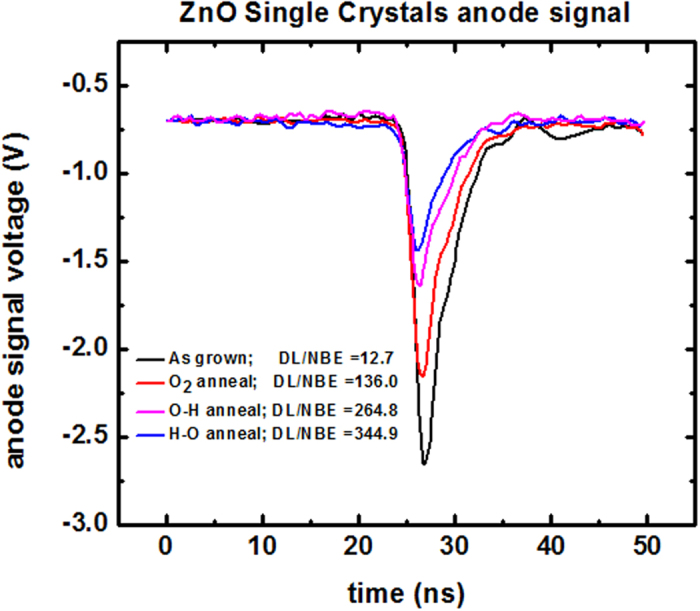
Fast-scintillation signal from ZnO single crystals coupled to a H3177-50 Hamamatsu photomultiplier tube.

The very fast decay of excitons in ZnO makes it a promising candidate for fast scintillation detectors. Here we investigate the effect of NBE and DL and on the scintillation signal from ZnO crystals. Figure 7 shows the output voltage of the anode signal from a photomultiplier tube coupled to as-grown and annealed ZnO crystals. The rise time of the signal for the as-grown crystal is about 1.3 ns, however it is limited by the rise time of the phototube. The graph includes the ratio between the DL and NBE results for each sample – as obtained from the XRIL measurements. This result reveals a strong correlation between this ratio and the amplitude of the scintillation signal from the anode - demonstrating how defects suppress the fast scintillation signal. These results are highly important for ZnO-based scintillators. As noted above, Ga-doped ZnO is a highly desirable candidate scintillator, in particular, for use as an α-particle detector for installation in a D-T neutron generator^42^,^43^. However, the nature of the luminescence centers and the energy-transfer mechanisms that are responsible for scintillation are not clearly understood^42^. The combined XRIL and scintillation measurements reported here provide information about the luminescence centers responsible for scintillation in ZnO and are expected to lead to improvements in the radiation detection performance of ZnO. In fact, the development of ZnO as a fast scintillator may be substantially advanced if one could control the defects and suppress their emissions.

## Conclusion

XRIL and PL were applied to study the NBE and DL values in high-quality ZnO single crystals grown by the CVT method. It is shown that XRIL allows a direct comparison to be made between the two emissions, and it can also be used to estimate the carrier recombination efficiency in ZnO. The GIPS technique was applied to defect investigations and indicated the presence of Zn vacancies passivated by hydrogen in all of the crystals. Accordingly, it is suggested that the green emission is associated with both oxygen vacancies and Zn vacancies passivated by hydrogen.

This work represents the first application of GIPS to semiconductors and shows that GIPS is a valuable positron-annihilation-lifetime spectroscopy technique for the characterization of defects in semiconductors since it eliminates the contribution from both the source materials and the surroundings. This is evident from the presence of a single lifetime component in the spectra. This technique will be particularly useful for the identification of additional defect types in semiconductors and dielectrics where standard PALS methods have limitations because of the source contributions. The use of γ-rays in GIPS measurements and X-rays in XRIL in this study allowed both the probing of defects and investigations of luminescence in the entire thickness of the crystals – i.e., studies that are not possible using standard techniques. The performance of semiconducting, optoelectronic, and photovoltaic devices are strongly affected by the presence and role of point defects and associated studies may benefit greatly from the usage of GIPS in characterizing defects. Lastly, the present scintillation measurements showed a direct correlation between the fast scintillation and the ratio between NBE and DL values. Overall, the results presented in this work provide a motivation to better understand, characterize and control defects in ZnO. They represent new and interesting methods for studies of defects and luminescence in ZnO and other materials.

## Methods

The un-doped ZnO single crystals investigated here were grown using the technique of chemical vapor transport (CVT). In this method, ZnO crystals grow directly from the vapor phase following the reduction of polycrystalline ZnO spheres that are positioned in a 5-cm diameter high-purity alumina ceramic tube. The ZnO crystal-growth process takes place via two steps where the first step consists of the use of flowing hydrogen gas to carry out the reduction of ZnO and, thereby, provide a concentration of zinc in the furnace position where the growth of bulk ZnO crystals occurs. This reduction process is given by: ZnO(s) + H_2_(g) → Zn(g) + H_2_O(g) (here “s” represents a solid and “g” a gas phase). This initial reaction takes place in a smaller diameter region of the overall two-stage alumina tube assembly at a position where the temperature is fixed at 1250 °C. An inert carrier gas such as nitrogen that is mixed with hydrogen is then used to transport the resulting Zn vapor to a cooler region of the growth furnace. In the second step, the crystal growth takes place in a chamber that is formed by a larger diameter tube of high-purity alumina and that is open to ambient air. In this final step, the crystals grow via the following reactions: Zn(g) + (1/2)O_2_(g) → ZnO(s) and H_2_ (g) + (1/2) O_2_(g) → H_2_O(g). The single crystals of ZnO that are grown by this CVT technique exhibit a prismatic habit with dimensions of up to several centimeters in length. For the current measurements, the crystals were cut to small pieces of approximately 10 × 7 × 1 mm^3^ and then annealed in two different environments: (1) flowing hydrogen at 300 °C and (2) flowing oxygen at 1100 °C. A few samples were annealed in both atmospheres – but in different order.

XRIL measurements were carried out using the newly developed spectrometer described in refs 16, 17. A Cu X-ray tube is used to generate X-rays that pass through a monochromator and collimator to provide a well-collimated mono-energetic X-ray beam that impinges on the sample. The light emitted from the sample is collected by a lens and transported through a polarized optical fiber to an Ocean Optics diffraction grating and CCD Sony detector. The luminescence spectra were recorded from 190 to 880 nm with a 0.4 nm resolution for a 65 second integration time. Focused visible light from a xenon source was used to align the light-collecting lens with the X-ray beam on the sample. The X-ray source, sample holder, and collecting lenses are placed inside a well- shielded chamber that blocks X-rays and also prevents external light from entering the chamber.

PL measurements were performed using a JY-Horiba FluoroLog-3 spectrofluorometer with double-grating excitation and emission monochromators with a 1200 groove/mm grating. The excitation source was a 450W CW Xenon lamp. Emission spectra were collected for the 325 and 390 nm excitation bands at room temperature. For the scintillation measurements, each ZnO single crystal was coupled to a Hamamatsu H3177-50 photomultiplier tube using optical grease and was wrapped by black tape to shield the phototube from external light. A negative 2300 V was applied to bias the phototube. The phototube was exposed to 662 keV γ-photons emitted from a Cs-137 source, and the scintillation signal from the anode was monitored using a 500 MHz oscilloscope.

PALS measurements were performed using the GIPS system at the superconducting electron accelerator facility, ELBE at HZDR in Dresden, Germany. The use of the superconducting electron accelerator with a high repetition rate (26 MHz) in the continuous wave (CW) mode permits the generation of intense high-energy γ-rays that produce a satisfactory number of positrons inside the sample through pair production. The created positrons thermalize, diffuse, and then annihilate. The accelerator generates very short pulses (5 ps temporal resolution) providing an excellent opportunity to perform PALS with good timing resolution in most materials. The measurements were carried out by a coincident measurement - Age Momentum Correlation (AMOC) - of the time of arrival and energy of the annihilation photons. This greatly reduces the background of scattered photons resulting in spectra with an excellent signal-to-background ratio^21^. The positron lifetime is registered as the time difference between the creation of positron in the sample (indicated by the accelerator pulse) and the detection of annihilation photons. In the current measurements, the electron-beam parameters were: pulse width 5 ps, current 600 μA, accelerator energy 16.9 MeV, repetition rate 26 MHz and a 12.5 μm thick Nb foil was used as a radiator to create the bremsstrahlung beam. Each ZnO sample was measured for 24 hours to obtain good statistics - since the volume of the sample is quite small. Two AMOC detection systems were employed to collect the annihilation photons from the sample at the same time to ensure the consistency of the results.

## Additional Information

**How to cite this article**: Ji, J. *et al*. ZnO Luminescence and scintillation studied via photoexcitation, x-ray excitation, and gamma-induced positron spectroscopy. *Sci. Rep.* **6**, 31238; doi: 10.1038/srep31238 (2016).

## Figures and Tables

**Figure 2:**
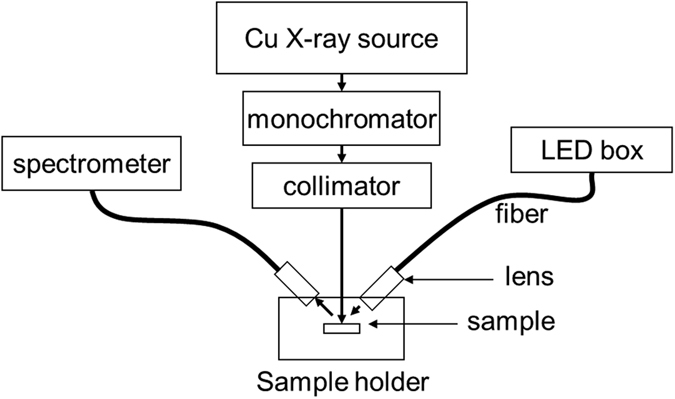
A schematic drawing of the XRIL/PL spectrometer which allows the use of both photo- and X-ray excitation.

**Table 1:** Positron lifetime data from GIPS measurements.

Probe	LT AMOC1 (ps)	Resolution AMOC1(ps)	LT AMOC2 (ps)	ResolutionAMOC2 (ps)	Chi-square AMOC1	Chi-square AMOC2
As grown	176.0 ± 0.2	160.2 ± 0.3	173.4 ± 0.2	184.4 ± 0.3	0.735	1.027
O_2_ anneal	174.9 ± 0.1	166.8 ± 0.2	172.8 ± 0.1	186.2 ± 0.3	1.003	1.312
H_2_ & O_2_ anneal	176.1 ± 0.2	159.7 ± 0.3	173.7 ± 0.2	181.2 ± 0.3	0.881	1.182
